# Determination of the Absolute Molar Mass of [Fe-S]-Containing Proteins Using Size Exclusion Chromatography-Multi-Angle Light Scattering (SEC-MALS)

**DOI:** 10.3390/biom12020270

**Published:** 2022-02-08

**Authors:** Christophe Velours, Jingjing Zhou, Paolo Zecchin, Nisha He, Myriam Salameh, Marie-Pierre Golinelli-Cohen, Béatrice Golinelli-Pimpaneau

**Affiliations:** 1 Fundamental Microbiology and Pathogenicity Laboratory, UMR 5234 CNRS-University of Bordeaux, SFR TransBioMed, 33076 Bordeaux, France; 2 Institute for Integrative Biology of the Cell (I2BC), CNRS, Université Paris-Saclay, 91198 Gif-sur-Yvette, France; 3 Laboratoire de Chimie des Processus Biologiques, UMR 8229 CNRS, Collège de France, Sorbonne Université, 11 Place Marcelin Berthelot, 75231 Paris, France; jingjing.zhou@college-de-france.fr (J.Z.); paolo.zecchin@college-de-france.fr (P.Z.); nshe1992@163.com (N.H.); 4 Institut de Chimie des Substances Naturelles, Université Paris-Saclay, CNRS, UPR2301, 91198 Gif-sur-Yvette, France; myriam.salameh@agroparistech.fr (M.S.); marie-pierre.golinelli@cnrs.fr (M.-P.G.-C.)

**Keywords:** SEC-MALS, size exclusion chromatography, multi-angle light scattering, molar mass, molecular weight, Fe-S cluster, iron–sulfur cluster, [Fe-S]-binding protein, NEET protein

## Abstract

Size Exclusion Chromatography coupled with Multi-Angle Light Scattering (SEC-MALS) is a technique that determines the absolute molar mass (molecular weight) of macromolecules in solution, such as proteins or polymers, by detecting their light scattering intensity. Because SEC-MALS does not rely on the assumption of the globular state of the analyte and the calibration of standards, the molar mass can be obtained for proteins of any shape, as well as for intrinsically disordered proteins and aggregates. Yet, corrections need to be made for samples that absorb light at the wavelength of the MALS laser, such as iron–sulfur [Fe-S] cluster-containing proteins. We analyze several examples of [2Fe-2S] and [4Fe-4S] cluster-containing proteins, for which various corrections were applied to determine the absolute molar mass of both the apo- and holo-forms. Importantly, the determination of the absolute molar mass of the [2Fe-2S]-containing holo-NEET proteins allowed us to ascertain a change in the oligomerization state upon cluster binding and, thus, to highlight one essential function of the cluster.

## 1. Introduction

Determining the molar mass (or molecular weight) is a critical step in the biophysical characterization of a newly produced protein. Analytical size-exclusion chromatography (SEC) is commonly used for the determination of the molar mass of proteins and protein–protein complexes in solution, based on the elution volume and a calibration curve based on protein standards [1]. However, when the protein is not globular or undergoes non-ideal column interactions, such determined molar mass is incorrect. In contrast, Multi-Angle Light Scattering (MALS) is an absolute technique that uses a collimated beam from a laser source to determine the exact mass in solution of proteins, lipids, detergents, nucleic acids, sugars or heterologous complexes and to evaluate their gyration radius [2,3,4,5]. The combination of SEC for separating proteins by their hydrodynamic radius, with MALS for determining the molar mass of the eluted protein, constitutes a versatile, reliable means for characterizing solutions of one or more protein species. Moreover, SEC-MALS, in which the measurement is performed at each elution volume, can determine if an eluting peak is homogeneous or heterogeneous and distinguish between a fixed molecular weight distribution versus a dynamic equilibrium [6,7]. Finally, this method enables the calculation of the stoichiometry of complexes such as protein–protein [6,8,9] or protein–carbohydrate complexes [10]. However, corrections of the light scattering measurements are required to determine the molar mass of polymers that are fluorescent or that absorb light at the operating laser wavelength. Whereas such corrections were previously described for lignin polymers [11], this topic is not frequently discussed in the literature. Thus, we address the case of iron–sulfur [Fe-S] cluster-containing proteins, which absorb light at the laser wavelength.

[Fe-S] clusters are among the oldest cofactors present in both aerobic and anaerobic organisms [12]. They are formed by ferrous and ferric ions bridged by sulfide ions. The most current forms are the [2Fe-2S] and [4Fe-4S] clusters [13], but more complex assemblies exist [14,15]. [Fe-S] clusters, typically coordinated by four cysteines, are known for their capacity to accept and give electrons, as exemplified by complex I of NADH quinone oxidoreductase [16]. The intrinsic chemistry of [Fe–S] clusters allows them to serve as sensors of gaseous (oxygen or NO) or nongaseous molecules (reactive oxygen species or ROS), or as a sensor of the Fe and [Fe-S] cluster content in the cell [17,18]. Numerous [Fe-S] proteins are also involved in regulating genetic expression [19,20]. For example, fumarate and nitrate reductase (FNR) is an interesting case of a transcription regulator, for which the oligomerization state of the protein changes with the chemical nature of the cluster [21,22]. Indeed, in the absence of O_2_, FNR contains a [4Fe-4S] cluster, which allows the dimerization of the protein, specific binding of the protein to DNA and transcription. Yet, in the presence of O_2_, the [4Fe-4S] cluster is rapidly converted into a [2Fe-2S] form, leading to the dissociation of the dimer and inactivation of FNR. Finally, some [Fe-S] proteins are enzymes, in which the [4Fe-4S] cluster serves as a cofactor to catalyze a chemical reaction [23], such as aconitase that catalyzes the dehydration of citrate [24,25].

Here we investigate [2Fe-2S]-containing regulatory proteins and [4Fe-4S]-dependent sulfuration and desulfidase enzymes. On the one hand, human CISD2 and mitoNEET are two mammalian NEET proteins anchored in the endoplasmic reticulum and mitochondria membranes, respectively, that possess [Fe-S]-containing cytosolic domains that are very close in sequence and structure [26]. This recently discovered class of [2Fe-2S]-proteins are characterized by atypical cluster coordination involving one histidine and three cysteines. Several studies have demonstrated their involvement in the regulation of iron and ROS homeostasis in cells [27]. One of their remarkable properties is their ability to transfer their cluster to a recipient protein under the strict control of the redox state of their cluster [28] and to be able to sense their environment [29]. On the other hand, sulfuration enzymes and desulfidases are [4Fe-4S]-dependent enzymes. tRNA sulfuration enzymes are involved in the biosynthesis of sulfur-containing nucleosides, which are essential for the efficiency and accuracy of genetic translation [30,31,32,33], whereas LarE catalyzes sulfur insertion in the cofactor of lactate racemase [34], TudS is a recently discovered thiouracil desulfidase [35] and CyuA is a cysteine desulfidase [36]. In the case of these sulfurases and desulfidases, the [4Fe-4S] cluster is bound by three ligands only, and the fourth non-protein bonded unique iron is presumed to bind and activate the sulfur donor for the sulfuration reaction [30,31] or the sulfur of the substrate for the desulfuration reaction [35].

The determination of the absolute molar mass is an important step, not only for the characterization of these [Fe-S]-binding proteins but also to reveal the potential involvement of the iron–sulfur cluster in protein oligomerization. In SEC-MALS, the SEC column is used solely to separate the various species according to their hydrodynamic radius so that they enter the MALS and concentration detector devices individually, the actual retention time not being significant for the analysis. The calibration of the instruments is independent on the column and does not rely on reference standards. Hence, SEC-MALS is considered an ‘absolute’ method to determine the molar mass from basic equations, using the ratio of the scattered and incident light intensities at several angles and the sample concentration determined by the refractive index (RI) detector. We detail here a correction method for analysis of SEC-MALS data of several [2Fe-2S]- and [4Fe-4S]-containing proteins, collected with the mini-DAWN TREOS Wyatt devices, that can be applied to any [Fe-S]-containing protein and, more widely, to any protein that absorbs at the laser wavelength. We show that the molar mass of the holo-proteins can be determined more accurately than without the correction and that, in the case of two NEET proteins, the binding of the cluster triggers the dimerization of the proteins.

## 2. Materials and Methods

Purification of human CISD2, *Escherichia coli* MnmA, *Aeromonas* TudS and human mitoNEET has been described previously [26,32,35,37], whereas the overexpression and purification of LarE and CyuA from *Methanococcus maripaludis* will be reported elsewhere (unpublished results). MitoNEET and CISD2 were purified aerobically as holo-proteins, whereas chemical cluster reconstitution was performed anaerobically with the as-purified proteins (mixture of apo/holo forms) of MnmA, TudS, LarE and CyuA, as described previously [32]. After anaerobic purification, these latter holo-proteins with labile [Fe-S] clusters were frozen and kept in air-free sealed tubes.

The as-purified MnmA, CyuA and LarE proteins contained such a low amount of cluster (<5%) that they were used as the apo-proteins. To obtain the apo-proteins of the NEET proteins, the cluster was removed by incubation of the holo-proteins in the presence of 10 mM ethylenediaminetetraacetic acid (EDTA) and 10 mM dithiothreitol (DTT) until the complete disappearance of the protein color and absorbance in the 320–600 nm range. Attempts to obtain the apo-form of TudS led to the aggregation of the protein.

As a final step of purification, all proteins were loaded on a gel filtration column (Hiload 26/60 Superdex 75 (Cytiva, Marlborough, MA, USA) for NEET proteins, Hiload 26/60 Superdex 200 (Cytiva) for all other proteins equilibrated with the same buffer as used for SEC-MALS analysis; the major eluted peak was collected. Including DTT at a high concentration in the final chromatographic step of the apo-proteins is essential to avoid the formation of intermolecular disulfide bonds between solvent-exposed cysteines.

Proteins were then analyzed with two different types of SEC-MALS/UV/RI light scattering equipment with very similar components: high pressure liquid chromatography (HPLC) from Agilent (Santa Clara, CA, USA) or Shimadzu (Tokyo, Japan), mini-DAWN TREOS and refractometer (Optilab T-rEX or Optilab), all from Wyatt Technology (Santa Barbara, CA, USA). For each sample, 100 µL at ~2 mg·mL^−1^ was injected on the HPLC system equipped with a Superdex 200 increase 10/300 GL column (Cytiva), at a flow rate of 0.5 mL·min^−1^ in 50 mM sodium phosphate pH 7.5, 50 mM NaCl, 10 mM DTT (for the [2Fe-2S] proteins) or 25 mM HEPES pH 7.5, 300 mM NaCl, 5 mM DTT (for the [4Fe-4S] proteins). The protein concentration was determined using an average refractive index increment (dn/dc) of 0.183 mL·g^−1^ at the laser wavelength (658 nm), in the 0.18–0.19 range used in classical buffers [38]. The light scattering detector was calibrated with toluene. The detector was controlled and the data were analyzed using the ASTRA 7.3 software (Wyatt Technology).

## 3. Theoretical Background

### 3.1. SEC-MALS Experiment

After separation of the species constituting the purified protein by HPLC on a SECcolumn and illumination by a laser with an incident intensity $I_{0}$ at a wavelength *λ*_0_ of 658 nm, the scattered light $I_{\theta }\;$ was recorded at three different angles. By measuring the excess Rayleigh ratio $R_{\theta }$ (the ratio of the scattered and incident light intensities at one measured angle *θ*), the concentration *c* by refractometry and adjusting the refractive index increment *dn/dc*, an absolute molar mass *M* could be obtained using Equation (1) [2,39]. This measurement is independent of the hydrodynamic radius of the macromolecule.

(1) $$R_{\theta }=K^{*}McP\left(\theta \right)\left[1-2A_{2}MP\left(\theta \right)c\right]\mathrm{with}\;K^{*}=4\pi^{2}{\left(dn/dc\right)}^{2}\frac{n_{0}^{2}}{N_{a}\lambda_{0}^{4}}\;$$

with *P*(*θ*), a corrective shape factor for big particles/molecules with respect to the laser wavelength [39],

(2) $$1/P\left(\theta \right)=1+q^{2}\frac{R_{G}^{2}}{3}$$

where *R_G_* is the gyration radius of the particle and *q* is the scattering vector given by [39],

(3) $$q=4\pi \;n_{0}\sin\left(\theta /2\right)/\lambda_{0}$$

*A_2_* is the second virial coefficient that is a corrective factor for a non-ideal solution and which accounts for the interaction between two particles/molecules, $n_{0}$, the refractive index of the solvent and *N_a_* the Avogadro number. In the case of online SEC-MALS, the $2A_{2}MP\left(\theta \right)c$ term in Equation (1) is negligible, the orders of magnitude of *A*_2_, *M* and *c* being 10^−4^, 10^5^ and 10^−3^, respectively. Using Equations (2) and (3), Equation (1) becomes:

(4) $$K^{*}c/R_{\theta }=\frac{1}{M}\;[1+(16\;\pi^{2}\;n_{0}^{2}\;R_{G}^{2}\;\sin^{2}\left(\theta /2\right)/3\;\lambda_{0}^{2})]$$

Experiments at several angles enable us to determine the molar mass, 1/*M* being the *y*-intercept of the Zimm plot $K^{*}c/R_{\theta }$ = f ($\sin^{2}\left(\theta /2)\right)$. A weighted linear fit of the data requires a minimum of three detectors covering a sufficient angular range.

**Figure 1:**
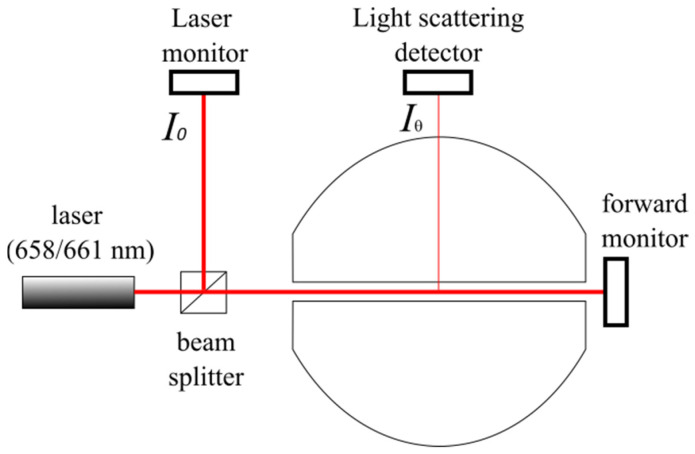
Schematic representation of the Mini-DAWN TREOS flow cell detectors (adapted from [40]).

Concretely, in the mini-DAWN TREOS system, the intensity of scattered light $I_{\theta }$ is measured by an array of photodiodes. The laser monitor measures the laser intensity $I_{0}\;$ before it enters the flow cell, whereas the forward laser monitor measures the transmitted light through the flow cell and sample (Figure 1). The excess Rayleigh ratio $R_{\theta }$ of scattered to incident light intensity needs to be corrected by the detector parameters (scattering volume *V* and distance of the scattering volume to the detector *r*), according to Equation (5) [40].

(5) $$R_{\theta }=\frac{I_{\theta }r^{2}}{I_{0}V}$$

An accurate measurement of the laser intensity, $I_{0},$ is essential for the determination of the molar mass by SEC-MALS. In the case of macromolecules without chromophores, the variations of the intensity of the laser source due to power fluctuations are taken into account by dividing each photodiode signal by that of the laser monitor.

### 3.2. Forward Monitor Correction Mode for Samples Absorbing at the Laser Wavelength

The Laser Monitor measures the intensity of the beam before it enters the cell, whereas the Forward Monitor measures the transmitted light through the flow cell and sample. This latter signal is used to determine the scattered intensity *I_corrected_* of absorbing samples. According to the Beer–Lambert law, the optical attenuation of the beam *F/F*_0_ due to the absorbing sample, measured by the Forward Laser monitor, depends on the optical path length through the sample *L*, its concentration *c* and its extinction coefficient *ε.*

(6) $$-\log(F/F_{0})=\varepsilon Lc$$

The scattered intensity at the center of the sample (*I_corrected_*) is related to the intensity of the incident laser *I*_0_ by:

(7) $$-\log(I_{corrected}/I_{0})=\varepsilon Lc/2$$

Combining Equations (6) and (7) leads to:

(8) $$I_{corrected}=I_{0}\sqrt{F/F_{0}}$$

Practically, to correct the absorption of the laser light by the samples, the ASTRA experiment file is opened with the ‘MALS TREOS Configuration’ and the ‘forward monitor’ mode is selected from the ‘Divide by Laser Monitor’ drop-down menu. The scattered intensity of the protein $I_{\theta }$ is obtained by subtracting the intensity scattered by the pure solvent from that of the total scattered intensity. This control is directly performed by the Astra software by subtracting the baseline ahead of the sample peak.

## 4. Results

We analyzed by SEC-MALS six [Fe-S]-binding proteins, two [2Fe-2S] regulatory proteins (CISD2 and mitoNEET) and four [4Fe-4S]-dependent enzymes (TudS, LarE, MnmA and CyuA) to obtain their molar mass in solution. Both the holo- and apo-forms were investigated for each protein, except for TudS, whose apo-form could not be obtained.

### 4.1. [Fe-S]-Binding Proteins Absorb at the Laser Wavelength

**Figure 2:**
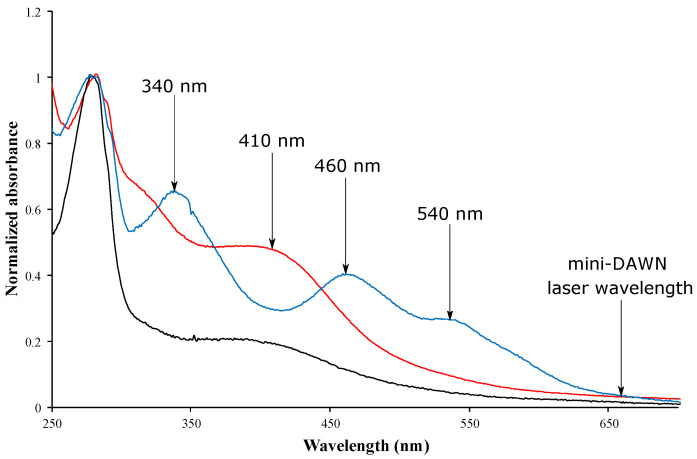
UV-visible absorbance spectra of the holo-forms of MnmA (black line), TudS (red line) and mitoNEET (blue line) between 250 and 700 nm. The absorption maxima for each holo-protein and the laser wavelength are indicated by arrows. Spectra were normalized using an absorbance value of 1 at 280 nm.

The absorption spectrum of [Fe-S]-binding proteins varies with the chemical nature of the cluster [41]. The apo-proteins absorb at 280 nm due to their aromatic amino acids but not in the visible light spectrum. In contrast, in addition to the peak at 280 nm, [4Fe-4S]-containing proteins exhibit a maximum of absorbance around 410 nm (Figure 2, red and black lines). The spectrum of [2Fe-2S]-containing proteins is more complex, with several characteristic absorption maxima between 320 and 430 nm, a peak around 470 nm and occasionally, a relatively broad peak between 550 and 600 nm (Figure 2, blue line). In both cases, the absorbance is not negligible at 658–661 nm, which corresponds to the laser wavelength.

### 4.2. Corrections for Samples Absorbing at the Laser Wavelength

Inaccurate molar masses are measured for macromolecules that absorb light at the laser wavelength (658/661 nm). In these cases, changes in measured intensity due to absorbance of the sample can be accounted for in the ASTRA software by dividing each photodiode signal by that of the forward laser monitor. Sample absorbance may be corrected by the procedure above if the forward monitor does not drop by more than approximately 20%. In addition, the effect of absorbance on the absolute molar mass determination can also be prevented by increasing the laser wavelength.

**Figure 3:**
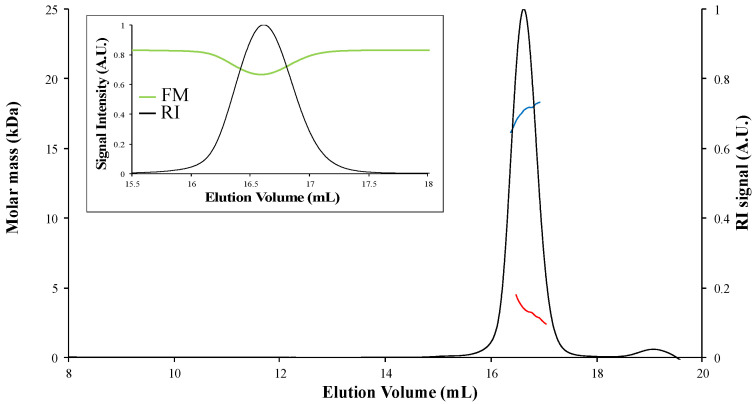
SEC-MALS analysis of holo-TudS. The molar mass was calculated using either the Forward Monitor (blue curve) or Laser Monitor correction modes (red curve). RI, refractive index; A.U., arbitrary unit. Insert: RI signal (black curve) and intensity signal of Forward Monitor detector (significative of protein absorbance; green curve), as a function of elution volume.

**Figure 4:**
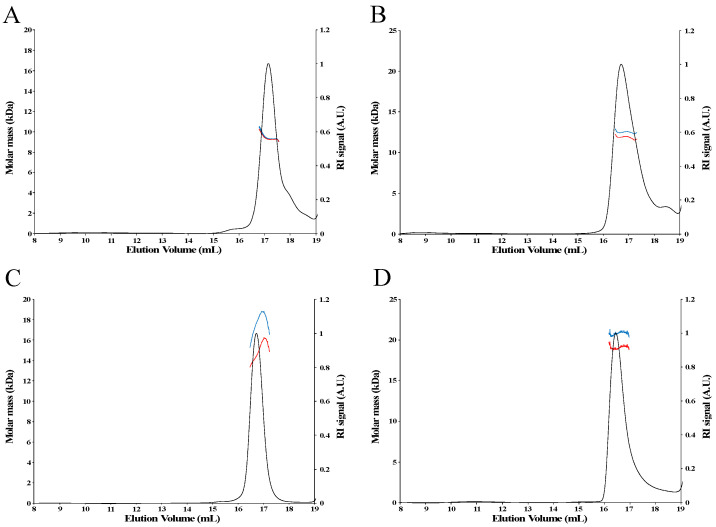
SEC-MALS analysis of apo- and holo-NEET proteins using either the Forward Monitor (blue curve) or Laser Monitor (red curve) modes. (**A**) apo-mitoNEET. (**B**) apo-CISD2. (**C**) holo-mitoNEET. (**D**) holo-CISD2.

For all proteins, the SEC-MALS chromatograms did not reveal peaks of high molar mass corresponding to aggregates (Figure 3 and Figure 4). In the presence of DTT, only one main peak was observed for the apo-proteins (Figure 4A,B and data not shown). For the holo-proteins, only one main peak was also observed, as exemplified in the case of holo-TudS (Figure 3) and the NEET proteins (Figure 4C,D), indicating no aggregation.

To calculate the molar mass of proteins from SEC-MALS data, one has to take into consideration the oligomerization state of the protein that is deduced by comparing the non-corrected experimental and theoretical molar mass values (Table 1). When analyzing the data of the studied apo-proteins that do not absorb light at the laser wavelength with the ASTRA software, the experimental molar masses determined without correction or using the Laser Monitor (LM) mode (normal mode for analysis of non-absorbing samples) correspond to the theoretical molar masses of the monomer or the dimer (Table 1). For the holo-proteins, the use of the Forward Monitor (FM) mode gives molar masses closer to the theoretical values than the LM mode, as shown by the significant variation in the experimentally determined molar mass, depending on whether the LM or FM modes were used. This difference was not observed with the apo-proteins, indicating that it originates from the presence of the [Fe-S] cluster and not from the protein sequence (Table 1). Therefore, the apo-proteins serve as a control (non-absorbing samples) for the validation of the method for correction of the absorbing samples. Regarding the holo-proteins, the variation of the molar mass calculated using LM or FM is exceptionally high for the TudS protein (80% decrease in molar mass compared to the theoretical value when LM is chosen) (Figure 3), significant for CISD2 and MnmA (close to 10%), and relatively low for LarE and CyuA (2–3%).

These variations seem to correlate with the differences between the theoretical value of the mass extinction coefficient at 280 nm (obtained using the ProtParam webserver https://web.expasy.org/protparam/ accessed on 2 February 2022) and the experimental value of the mass extinction coefficient obtained using the ASTRA “UV -extinction from RI peak” method (Table 2). For the apo-proteins, the experimental and theoretical extinction coefficients values are very close to each other, which is not the case for the holo-proteins. In particular, the huge difference between the theoretical and experimental extinction coefficients for holo-TudS remains unexplained.

## 5. Discussion

Our SEC-MALS analysis indicates that both human NEET proteins, mitoNEET and CISD2, are monomeric in their apo-forms but dimeric in their holo-forms. We thus confirm here that the [2Fe-2S] cluster is essential for the stabilization of the dimer of both NEET proteins. We had previously shown that the cluster is essential for the folding of the mitoNEET [42] and CISD2 [26] protomers. Indeed, the loss of the cluster leads to incorrect folding of the protomer and, consequently, to the loss of protein dimerization. Moreover, native mass spectrometry showed that holo-mitoNEET is a dimer but not apo-mitoNEET [29]. It is interesting to note that although the [2Fe-2S] cluster of the two NEET proteins is essential for dimerization, it is not located at the interface between the monomers, as shown by the crystal structures of mitoNEET [43,44,45] and CISD2 [46]. In contrast to the NEET proteins, LarE and CyuA are intrinsically dimeric because both the apo-and holo-forms are dimers, indicating that the cluster is not crucial for the correct folding of these proteins.

This study reveals the power of SEC-MALS to investigate the involvement of an [Fe-S] cluster in maintaining the oligomerization state of a protein. The binding of a [2Fe-2S] cluster at the dimer interface has been reported for several proteins. Human mitochondrial ISCU2 is the scaffold protein of the ISC (iron–sulfur cluster assembly) complex involved in the synthesis of [Fe-S] clusters in living cells and the subsequent transfer of clusters to target apoproteins. ISCU2 synthesizes a bridging [2Fe-2S] cluster by assembling two ISCU2 monomers. It has recently been explained how, after iron and sulfur loading onto monomeric ISCU2, the interaction between tyrosines, belonging to two different monomers, triggers ISCU2 dimerization and the generation of a bridging [2Fe-2S] cluster [47]. Similarly, human cytosolic monothiol glutaredoxin-3 (GLRX3) is a protein essential for the maturation of cytosolic [4Fe-4S] proteins. Dimeric cluster-bridged GLRX3 transfers its [2Fe-2S] clusters to human NUBP1, an essential early component of the cytosolic iron−sulfur assembly (CIA) machinery [48]. Determining the molar mass of both the apo- and holo-forms of such proteins is essential to shed light on the structural function of the cluster.

## 6. Conclusions

SEC-MALS is a valuable technique to determine the molar mass in solution of proteins in their native state, which does not require desolvation of the sample or very low salt concentrations. The Wyatt miniDAWN TREOS instrument can be used to determine the absolute molar mass of proteins, even when they exhibit absorption at the laser wavelength, as is the case for the [Fe-S]-containing proteins discussed in this article, by using the Forward Monitor instead of the Laser Monitor mode. At present, the MALS devices on the market, Viscotek TDA 305 and OMNISEC REVEAL from Malvern Panalytical (Malvern, United Kingdom) or LenS3 from Tosoh Bioscience (King of Prussia, PA, USA), do not provide the possibility to correct for absorption of samples at the laser wavelength. Obtaining the holo-form of these proteins is often difficult because the cluster can be easily destroyed by oxygen, so working under anaerobic conditions is required. In our study, the holo-proteins with labile clusters were purified and frozen anaerobically, and SEC-MALS analysis was performed aerobically immediately after thawing. Whereas it is tempting to determine only the molar mass of the apo-protein, we show here that also determining the molar mass of the holo-protein is crucial for understanding potential changes in oligomerization states upon cluster binding, which may be crucial for the function of the protein, as shown in the case of the [2Fe-2S]-containing NEET proteins.

## Figures and Tables

**Table 1:** Variation of the experimentally determined molar mass using the LM and FM modes.

		Experimental Molar Mass						
Protein	Theoretical Molar Mass (kDa) ^1^		No Correction (kDa)	Oligomerization State ^1^	LM ^2^ (kDa)	Mass Difference with Theoretical Value Using LM ^3^ (%)	FM ^2^ (kDa)	Mass Difference with Theoretical Value Using FM ^3^ (%)
mitoNEET	8.6 (M); 17.2 (D)	Apo	9.3	M	9.3	−8.1	9.3	−8.1
		Holo	16.2	D	16.2	5.8	18.8	9.3
CISD2	11.4 (M); 22.8 (D)	Apo	11.8	M	11.9	−4.4	12.6	−10.5
		Holo	19	D	19	16.7	20.9	8.3
MnmA	40.6 (M)	Apo	36.3	M ^4^	36.3	10.6	36.3	10.6
		Holo	36.1	M	36.5	10.1	40.1	1.2
LarE	31.1 (M); 62.2 (D)	Apo	54.1	D	54.2	12.9	54.2	12.9
		Holo	58.8	D	58.8	5.5	60.5	2.7
CyuA	43.7 (M); 87.4 (D)	Apo	77.9	D	77.9	10.9	78.6	10.1
		Holo	78.9	D	78.9	9.7	80.8	7.6
TudS	16.5 (M)	Holo	4.5	M	3.3	80.0	17.9	−8.5

^1^ M, monomer; D, dimer. ^2^ Molar mass determined using the Laser Monitor mode (normal mode) or the Forward Monitor mode (that corrects for absorbing samples). ^3^ [monomer theoretical value × N − LM (or FM) value]/ monomer theoretical value × N, with N = 1 (monomer) or N = 2 (dimer). ^4^ 4.8% dimeric form was observed in the presence of 5 mM DTT. In the absence of DTT, the % dimer increased to 49.1% [32].

**Table 2:** Comparison of the theoretical and experimental extinction coefficients at 280 nm for the six studied [Fe-S] proteins.

Protein Name		Theoretical Extinction Coefficient mL/mg·cm	Experimental Extinction Coefficient mL/mg·cm
mitoNEET	Holo	0.815	1.545
	Apo		0.846
CISD2	Holo	0.616	1.2262
	Apo		0.768
MnmA	Holo	0.993	1.46
	Apo		1.1
LarE	Holo	0.473 ^1^	0.638 ^2^
	Apo		0.565
CyuA	Holo	0.583	0.858
	Apo		0.733
TudS	Holo	0.514	1.406

^1^ no tryptophan; ^2^ the experimental extinction coefficient value at 280 nm was deduced from the integration of the peak at 410 nm in the UV-visible spectrum.

## Data Availability

Not applicable.
